# Extraction efficiency and bioactive evaluation of *Tamarix nilotica* and *Arthrocnemum macrostachyum* extracts for anti-cancer potential

**DOI:** 10.1371/journal.pone.0311567

**Published:** 2025-01-21

**Authors:** Maryam Al Kaabi, Nancy A. ElNaker, Nila Jan, Michael A. Ochsenkühn, Shady A. Amin, Lina F. Yousef, Ahmed F. Yousef

**Affiliations:** 1 Department of Chemistry, Khalifa University, Abu Dhabi, United Arab Emirates; 2 Marine Microbiomics Lab, New York University Abu Dhabi, Saadiyat Island, Abu Dhabi, United Arab Emirates; 3 Department of Biological Sciences, Khalifa University, Abu Dhabi, United Arab Emirates; 4 Center for Membranes and Advanced Water Technology (CMAT), Khalifa University, Abu Dhabi, United Arab Emirates; 5 Center for Biotechnology (BTC), Khalifa University, Abu Dhabi, United Arab Emirates; BOKU: Universitat fur Bodenkultur Wien, AUSTRIA

## Abstract

This study aimed to evaluate the potential of phytochemicals from two native UAE plant species, *Arthrocnemum macrostachyum* and *Tamarix nilotica*, as anti-cancer agents. The plant extracts were obtained using two methods, maceration, and microwave-assisted extraction (MAE), and were subsequently evaluated for their in vitro cytotoxicity against three cancer cell lines: breast (MDA-MB-231), colon (HCT-116), and lung (A-549). Results suggest that: 1) MAE is more efficient than maceration in recovering metabolites from plant biomass based on measurements of total phenolic content, radical scavenging activity, and bioactivity of extracts based on in vitro cytotoxicity. 2) Only *T*. *nilotica* extracts were found to be bioactive based on cytotoxicity measurements. 3) Cancer cell lines displayed differential sensitivity to *T*. *nilotica* crude extracts, with breast cancer cells being the most sensitive and lung cancer cells being the least sensitive. 4) Solid-phase fractionation of *T*. *nilotica* crude extract using different percentages of methanol resulted in several fractions that were 100-fold more cytotoxic compared to the crude unfractionated extract. The 30% and 70% methanol fractions exhibited the highest cytotoxicity towards breast and colon cancer cell lines, respectively. 5) Untargeted metabolomics using UHPLC-Q-ToF-MS of *T*. *nilotica* crude extracts revealed 909 molecular features, of which only 327 were annotated using MS/MS fragmentation. The results suggest that *T*. *nilotica* extracts have potential as anti-cancer agents and that MAE is an efficient method for extracting phytochemicals from plant biomass. The study also revealed that cancer cell lines exhibited differential sensitivity to the extracts and that solid-phase fractionation of crude extract using different percentages of methanol can yield fractions that are more cytotoxic than the crude extract.

## Introduction

Cancer is a broad group of diseases characterized by the uncontrolled growth of cells in the body, which compromises the body’s overall function [1,2]. Despite significant advancements in treatments and preventative therapies, cancer remains a major cause of illness and death worldwide. In the UAE, cancer has been the second leading cause of non-communicable diseases (NCD)-related mortality [3]. As documented in the GLOBOCAN 2012 reports, the incidence rate of cancer reached 92.5, and the mortality rate is 58 per 100,000 people [4,5]. Among women, breast cancer is the most prevalent type, accounting for 43% of cancers diagnosed in women and 25% of all cancer cases in the UAE [6]. Women in the UAE tend to develop this disease a decade earlier than women in Western countries [7]. On the other hand, lung cancer is the most common type among men [5].

Current cancer treatment regimens include various methods such as surgery to remove tumors, chemotherapy, immunotherapy, radiotherapy, photodynamic therapy, and stem cell transformation [8]. However, these treatments have limitations such as the adverse side effects on non-targeted tissues and toxicity in healthy tissues [1,8]. Therefore, there is a constant need for alternative treatments and therapies against cancer. Plant-derived mixtures are considered a promising source for these alternative treatments [1]. The development of natural products has been the foundation for sophisticated traditional medicine [9–11]. It’s worth mentioning that over 60% of anticancer agents currently in use are derived from natural sources such as plants, marine organisms, and microorganisms [12,13]. Natural products have been widely used in cancer treatments for many years. Although new and synthetic drugs are developed through combinatorial chemistry, natural products offer a diverse range of compounds that have promising biological properties. This success in drug discovery is due to the high chemical diversity of natural products [14]. However, the detection and characterization of these compounds can be challenging because crude extracts contain many compounds that are chemically diverse. Traditional methods of discovering new active compounds have several limitations, such as complexity and long timeframes. Therefore, faster separation and isolation steps are needed to identify the active compounds from crude extracts. Natural product screening is a recent technique that has become advantageous for rapidly selecting metabolites with biological properties [9,11,15].

A reliable and sophisticated screening system is required to detect the target substance in a mixture of compounds with high specificity and sensitivity [16,17]. Metabolomics provides an appropriate and convenient approach by performing an uninterrupted analysis of the crude extract and detecting a broad spectrum of metabolites that belong to various chemical classes, avoiding time-consuming isolation procedures [9,18,19]. It is a comprehensive method to identify all endogenous metabolites in a given organism or biological sample. This approach improves the effectiveness of previous methods and restores the importance of natural products as a source of potential anticancer agents [20,21].

Secondary metabolites in the plant kingdom, such as polyphenols, flavonoids, and brassinosteroids, have been studied for their potential use as anticancer agents. They have been found to possess anticancer activities such as antioxidant activity, blocking cancer cell growth, inducing apoptosis, target specificity, and cancer cell cytotoxicity [22–25]. Cytotoxicity screening models are essential methods for the selection of active plant extracts against cancer. In this study, a new screening method was applied to explore secondary metabolites from two native halophyte species (*Tamarix nilotica* and *Arthrocnemum macrostachyum*) that could be promising leads for the development of novel anticancer agents. The most active fractions obtained from this separation were further fractionated based on their cytotoxicity against three different types of human cancerous cell lines: human breast (MDA-MB-231), human colorectal adenocarcinoma (HCT-116), and human lung adenocarcinoma (A-549). Additionally, the metabolic profiling of the crude aqueous extracts of these halophytes was conducted using Ultra-High-Performance Liquid-Chromatography coupled with a quadrupole time-of-flight mass spectrometer (UHPLC-Q-ToF-MS). *T*. *nilotica* (Ehrenb) is a local halophyte found in the UAE, and its extracts have been reported to possess several biological activities related to their phenolic content, such as hepatoprotective, antioxidant, antiangiogenic, antidiabetic, antifibrotic, antimicrobial, and anticancer activities [26–28]. On the other hand, *A*. *macrostachyum* is a halophyte that grows along the Arabian Gulf of the UAE and is known for its small and fleshy leaves. This halophyte has also been used in ethnomedical applications for its antioxidant, anti-inflammatory, antidiabetic, and antimicrobial activities [29].

Therefore, the goal is to identify the potential anti-cancer compounds in the two halophytes and to compare their metabolomics profiles. The main objectives of this study are: 1) To evaluate the *in vitro* cytotoxicity of plant crude aqueous extracts from two halophytes (*T*. *nilotica* and *A*. *macrostachyum*) against three cancer cell lines; MDA-MB-231, HCT-116, and A-549. 2) To determine and compare the metabolomics profile of crude aqueous extracts prepared from two UAE native halophytes using UHPLC- Q-ToF-MS. 3) To use a bio-guided fractionation approach to obtain purified fractions that contain the bioactive compounds originally present in the complex crude plant extracts.

## Results and discussion

### Extraction and comparison between chosen methods

Two methods of extraction: maceration, and MAE were performed and compared in terms of TPC (measured as mg of Gallic acid equivalent (GAE) per g of dry extract), antioxidant capacity (measured as IC50 of DPPH radical in μg/mL), and MDA-MB- 231 cytotoxicity (measured as LD50 in μg/mL) for the two plants, *T*. *nilotica* and *A*. *macrostachyum* as shown in Table 1.

**Table 1:** Effect of varying extraction techniques on breast cancer cytotoxicity (LD50), half-maximal inhibitory concentration (IC50) for DPPH-free radical, and total phenolic content (TPC) of crude extracts from *Tamarix nilotica* (*Tn*) and *Arthrocnemum macrostachyum* (*Am*). Values are means ± standard error from three independent experiments.

*Crude extract*	*Extraction method*	*LD50 (μg/ml)*	*IC50 (μg/ml)*	*TPC (mg GAE/g dry extract)*
*Tamarix nilotica*	Maceration	1735 ± 0.016	88.6 ± 0.055	18.5 ± 0.06
	MAE	653.3 ±0.038	53.1 ± 0.041	31.1 ±0.0015
*Arthrocnemum macrostachyum*	Maceration	> 5000	95.0 ±0.0052	17.4 ± 0.529
	MAE	3397 ± 0.032	60.61 ± 0.05	28.7 ± 0.1

Two general observations are made upon collective comparison of data presented in Table 1. The first observation is that MAE is superior to maceration because MAE extracts from both plants contained almost 2-fold higher amounts of TPC and appear to be more bioactive when compared to maceration extracts (Table 1). The second observation is *T*. *nilotica* extracts appear to exhibit higher bioactivity when compared to *A*. *macrostachyum*. An extract is considered bioactive if the cytotoxicity LD50 is equal to or less than 100 μg/mL [30]. According to this cut-off threshold, none of the crude extracts are bioactive (Table 1). However, there is almost a 5-fold difference in LD50 cytotoxicity between the two plant extracts regardless of which extraction method was used (e.g., for MAE, the LD50 is 653 Vs 3397 μg /mL for *T*. *nilotica* and *A*. *macrostachyum*, respectively). The differences in bioactivity reported for the two plant species does not appear to be related to TPC because similar amounts are found in the extracts regardless of the extraction method used; for example, in MAE extracts, TPC is approximately 31 and 29 mg GAE/g dry extract for *T*. *nilotica* and *A*. *macrostachyum*, respectively.

The TPC reported in this study is in agreement with previous reports estimating TPC of *T*. *nilotica* butanol extracts (22.12 ± 2.4 mg/g GAE) [31] and TPC of *A*. *macrostachyum* diethyl ether extracts from plants collected from Southern Portugal (33 mg GAE/g dry extract) [32]. In general, the TPC of both plants is low compared to other economically important plants such as oak (*Quercus robur*), pine (*Pinus maritime*), and cinnamon (*Cinnamomum zeylanicum*), which ranged from 300–400 mg GAE/g [33].

The dose-dependent antioxidant activity was observed in all extracts regardless of the extraction method used. Among all, crude fraction from MAE *T*. *nilotica* displayed the highest antioxidant activity (IC50 = 53.1 ± 0.041 μg/mL) (Appendix E illustrate the dose-response curve for this sample). Furthermore, there is a strong positive correlation (r^2^ = 0.995, see Appendix F) between IC50 of DPPH radical scavenging activity and TPC, confirming a function for phenols as antioxidants or radical scavengers. The antioxidant activity of *T*. *nilotica* extract could be attributed to the phytochemicals present, mainly phenolic compounds. Hence, the variation in antioxidant capacity between the two types of extraction is perhaps related to the difference in their phenolic content [34], which is affected by the extraction method [35]. Moreover, due to the direct relationship between antioxidant and anticancer activity, higher levels of antioxidant compounds—such as polyphenols, carotenoids, and tannins—can enhance cytotoxic effects. These antioxidants are vital for human health as they neutralize free radicals and chelate toxic substances [28]. For this reason, an anticancer activity analysis was conducted on the crude plants extract.

### Sensitivity of the cell lines to crude plant extracts

**Fig 1:**
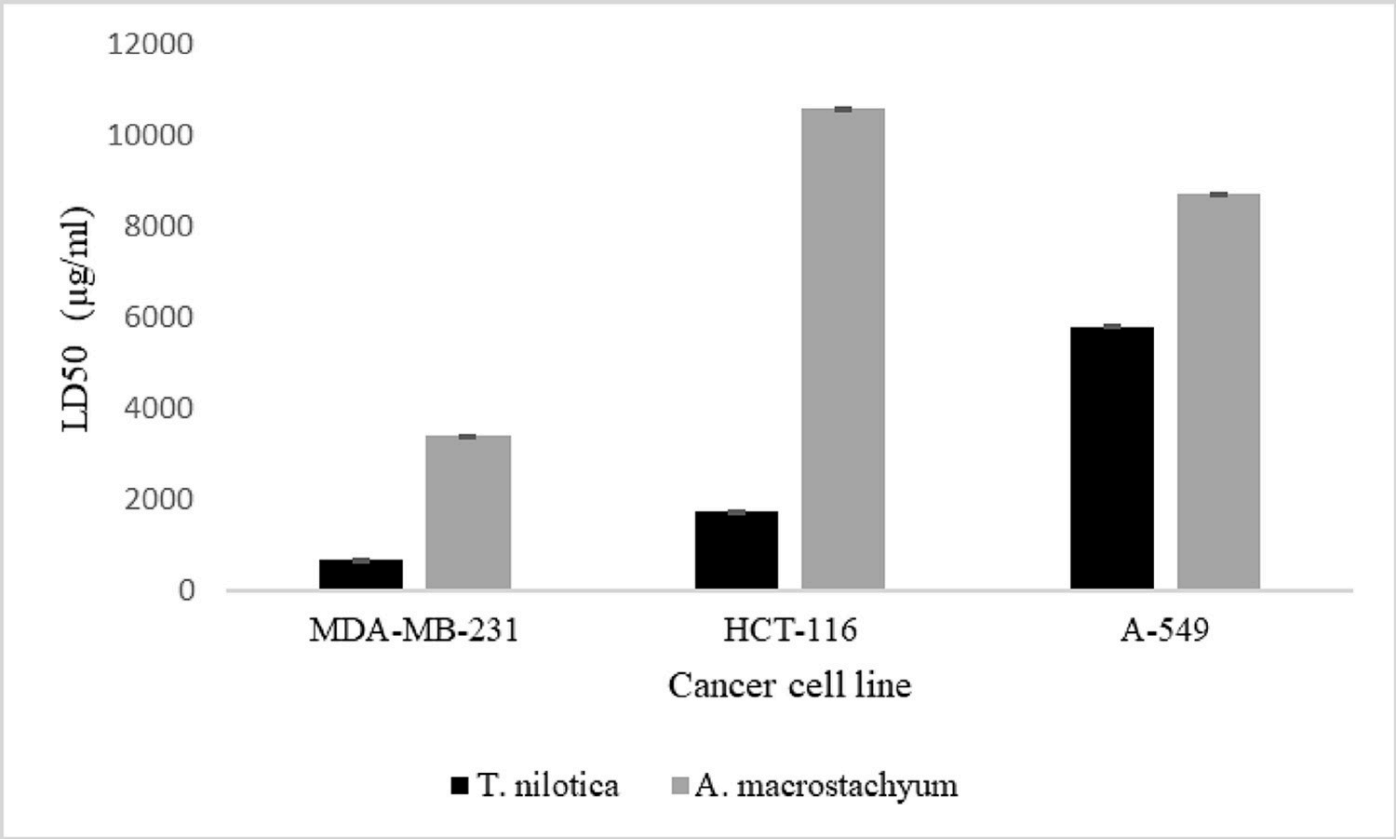
Cytotoxicity of crude extracts from *Tamarix nilotica* and *Arthrocnemum macrostachyum* towards three human cancer cell lines; breast cancer cell line (MDA-MB-231), colon cancer cell line (HCT-116), and lung cancer cell line (A-549). Results are expressed as LD50 values (μg/mL). Values are means ± standard error from three independent experiments.

Following the initial comparisons, only the MAE extracts were evaluated in further investigations due to its superior efficiency compared to the conventional maceration extraction method. The crude plant extracts were tested for their ability to inhibit the growth of three different types of cancer cells: breast cancer MDA-MB-231, colon cancer HCT-116, and lung cancer A-549 cell lines using the MTT assay (Fig 1).

There were no or very little toxic effects observed from *A*. *macrostachyum* extracts on all three cell lines, which supports the traditional usage of this species for cattle feed and treating poisonous bites and stings, indicating its low toxicity [36]. On the other hand, *T*. *nilotica* extracts showed stronger activity between the cell cultures, with LD50 values of 653.3 ±0.038 μg/mL for MDA-MB-231 and 1717.0 ±0.087μg/mL for HCT-116. Previous research found that *Tamarix Nilotica* butanolic fractions showed promising anticancer activity against liver cancer cell Huh-7 with IC_50_ = 37 ug/ml [27]. Several macrocyclic-type tannins isolated from *T*. *nilotica* showed significant cytotoxicity against carcinoma cell lines, including oral squamous cell carcinoma and promyelocytic leukemia, while demonstrating lower cytotoxicity towards normal cells, suggesting their potential for development into effective antitumor agents with relatively low toxicity [28]. As a result of this, only the crude extract of *T*. *nilotica* was fractionated in different concentrations of methanol to obtain bioactive fractions (LD50 <100 μg/mL), as discussed below.

### Metabolomics of crude fractions from *T*. *nilotica* and *A*. *macrostachyum*

**Fig 2:**
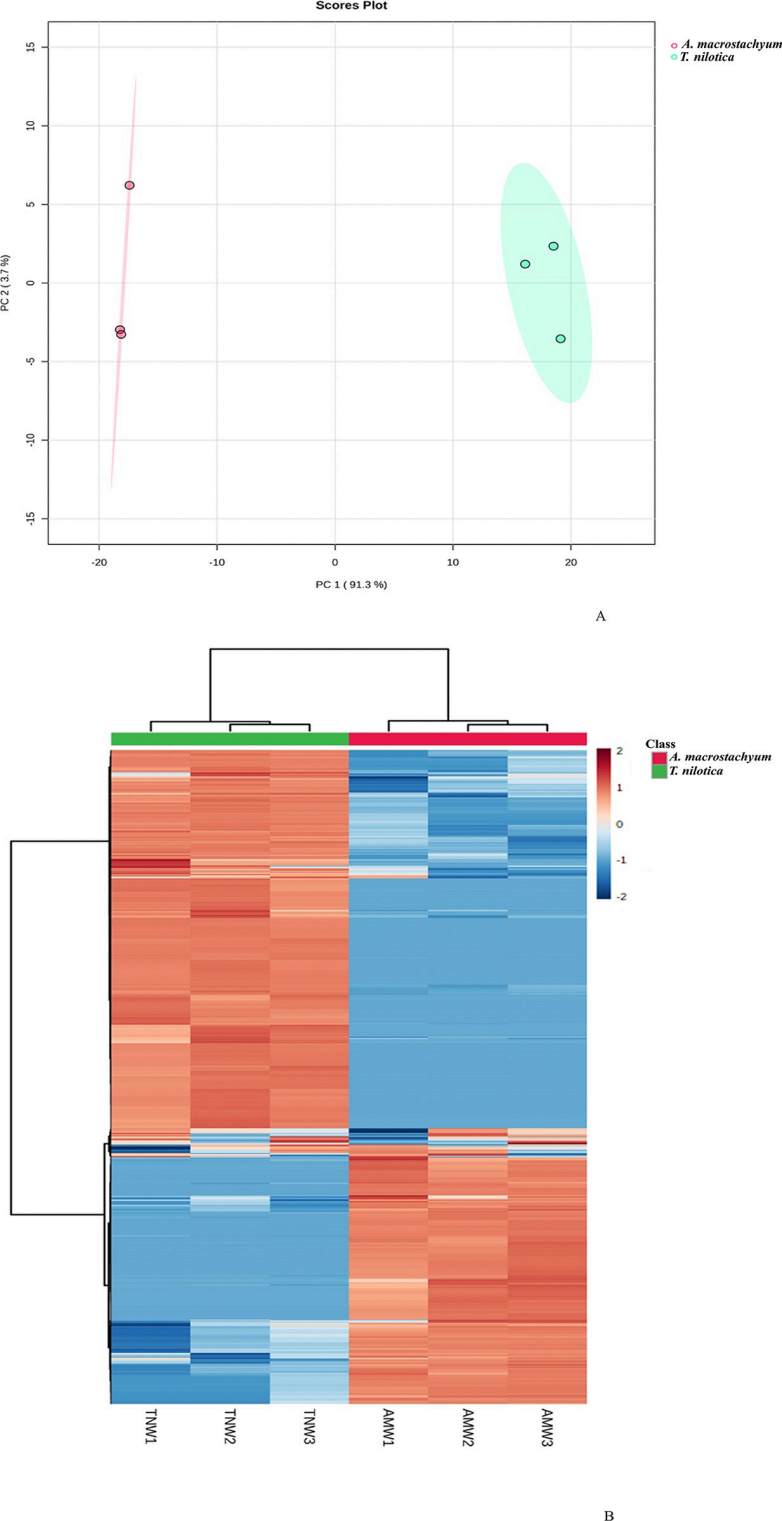
A) Graphical representation with 2-D scores of two principal components PC 1 and PC 2 comparing crude extracts of *Tamarix nilotica* (green circles) and *Arthrocnemum macrostachyum* (red circles). Each sample plant has three experimental replicates. PCA was carried out after data log transformation and Pareto scaling. B) Heat map dendrogram plots of the metabolites analyzed by UPLCQ-TOF MS in crude extracts from *Tamarix nilotica* (green label), and *Arthrocnemum macrostachyum* (red label) plants. Each plant species has three experimental replicates; *Tamarix nilotica* water extracts (TNW1, TNW2 and TNW3) and *Arthrocnemum macrostachyum* water extracts (AMW1, AMW2 and AMW3). Heatmap was carried out using Euclidean distance and clustering algorithm using ward.

To compare the metabolomics profile of the crude aqueous extracts of the two plant species *T*. *nilotica* and *A*. *macrostachyum*, UHPLC-Q-ToF-MS was performed. This approach was also adopted to identify the specific metabolites in *T*. *nilotica* that have been reported to have anticancer bioactivity in previous studies. A total of 1336 molecular features were detected using UHPLC-Q-ToF, and out of those, 909 molecular features were found to have significant differences in abundance when comparing the two plant extracts. Of the 1336 molecular features, only 307 were identified using MS/MS fragmentation. Out of these identified compounds, 239 were found in *T*. *nilotica* extract, and 187 were found in *A*. *macrostachyum*. The principal component analysis (PCA) of the molecular features shows a clear separation of two distant clusters (PC1–91.3% and PC2–3.7%) based on plant identity (Fig 2A). The three experimental replicates from *A*. *macrostachyum* extracts clustered more tightly compared to *T*. *nilotica* extracts (Fig 2A) suggesting slight variability in one of the experimental replicates from *T*. *nilotica*. Overall, the PCA plot shows a statistical distinction in metabolite chemical composition of *T*. *nilotica* and *A*. *macrostachyum* crude extracts. Similarly, the clustered heatmaps also demonstrate substantial differences in chemical composition between the *T*. *nilotica* and *A*. *macrostachyum* plant extracts (Fig 2B). The dendrogram depicted above the heatmap also reveals that there is some variability among the experimental replicates for an extract from a plant species.

### Phytochemical profile of *T*. *nilotica*

**Fig 3:**
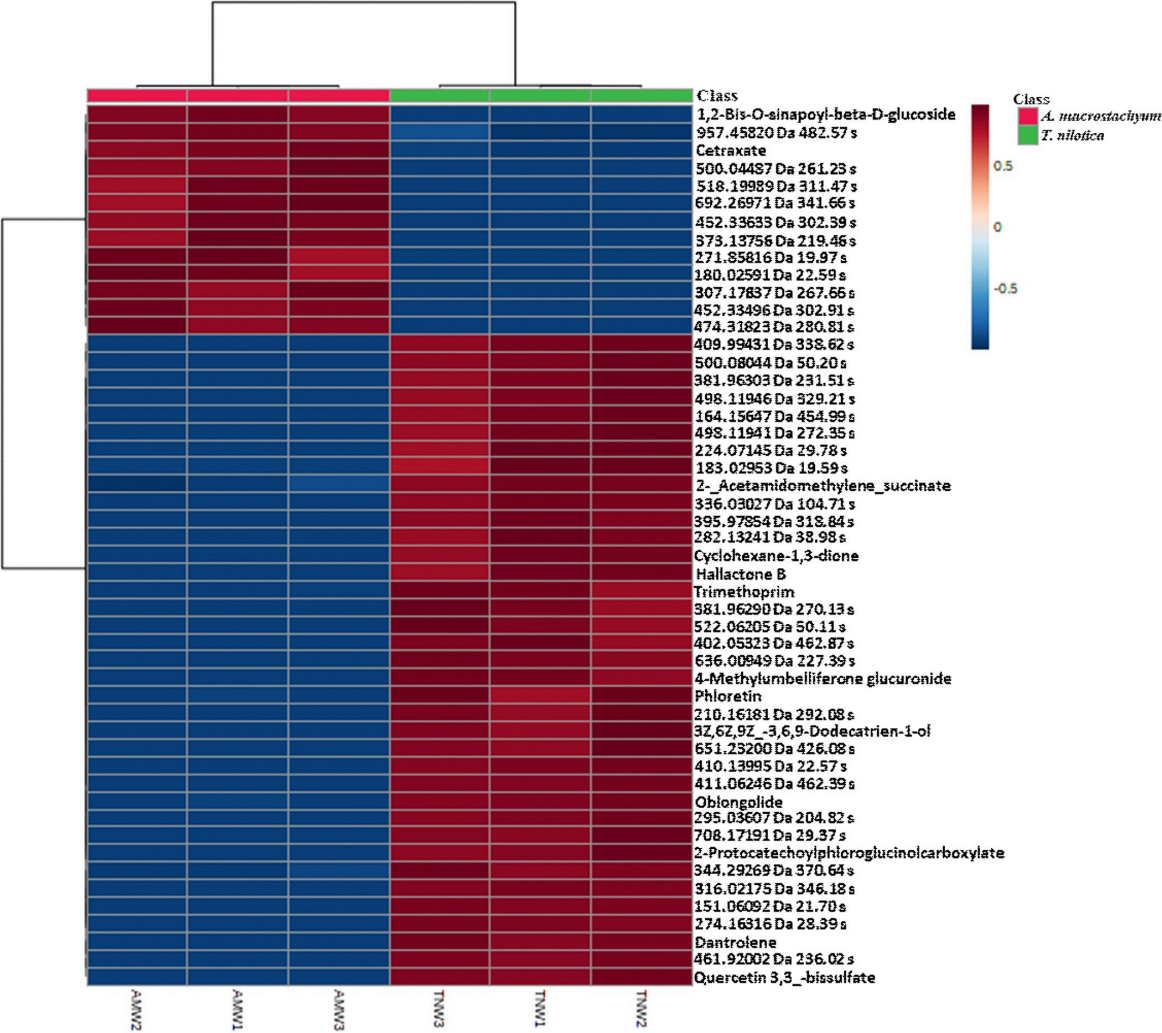
Clustered heatmap plot of top 50 molecular features (differences detected using ANOVA (P = 0.05) in crude extracts from *Tamarix nilotica* (green label) and *Arthrocnemum macrostachyum* (red label) plants. Each plant species has three experimental replicates. Heatmap was obtained using Euclidean distance and clustering algorithm using ward.

Fig 3 shows a heatmap of the top 50 molecular features found in *A*. *macrostachyum* and *T*. *nilotica* crude extracts. Out of these 50 features, only 11 of them can be identified in the *T*. *nilotica* extract. These 11 metabolites are classified as terpenoids, flavonoids, coumarins, salt, sugars, or other compounds. Most of these features are terpenoids, which are known for their bioactive properties and are commonly used in medicine, particularly as anticancer drugs like Taxol and its derivatives. The two main terpenoids compounds found in *T*. *nilotica* are triptolide and hallactone B. The flavonoids identified in the extract are quercetin 3,3-bissulfate and 2-protocatechoylphloroglucinolcarboxylate, which is a derivative of quercetin. Flavonoids are known to have various biological activities and are used in cosmetic, nutraceutical and pharmaceutical products [37]. Quercetin has been shown to inhibit the growth of various types of human cancer cells through its pro-apoptotic effects. Another compound found in *T*. *nilotica* is 4-methylumbelliferone sulfate, which is classified as a coumarin. This compound has been found to have inhibitory effects on multiple types of cancer, including skin [38,39], breast [40], liver [41], prostate [42], pancreatic [43], and bone cancer [44–46].

Table 2 shows other molecular features that have been previously reported in studies of *T*. *nilotica* and Table 3 shows a list of compounds that have been reported to have bioactive properties such as antitumor, anti-inflammation, antimicrobial and enzyme inhibitory activity in other studies [47], and were also detected in the metabolomic profile of *T*. *nilotica* extract in this study. These Tables 2 and 3 also list the computed XlogP3 values for each molecule from PubChem’s chemical database. The logP values of the molecules found in *T*. *nilotica* extract range from -1.7 for citrate to 9.6 for Lupenon (Table 3). These logP values indicate that some of the molecules in the *T*. *nilotica* extract may not be very water-soluble (XlogP3 > 1), which could affect their recovery when using water as an extraction solvent. To optimize the recovery of bioactive metabolites from *T*. *nilotica*, it may be worth exploring the use of organic solvents instead of water for the extraction process in future experiments.

**Table 2:** List of annotated molecular features detected in *T*. *nilotica* crude extract were also reported in other studies. The table shows retention time (RT) and mass-to-charge ratio (*m/z*) obtained from UHPLC-MS in this study, and XlogP3 values obtained from PubChem’s online chemistry database.

Num.	Compound name	RT	*m/z*	XlogP3	Reference
1	Quercetin-3-glucoside	5	465.10284	0.4	El-Sisi *et al*., 1973
2	Gallic acid	0.56	171.02821	0.7	Nawwar *et al*., 1982
3	Isorhamnetin 3-glucoside	4.86	479.1186	0.7	Ren *et al*., 2019
4	Quercetin 3,3-bissulfate	4.03	462.96351	1	Nawwar *et al*., 1984
5	Ellagic acid	4.14	303.01323	1.1	Nawwar *et al*., 1984
6	Caffeic acid	4.68	343.1023	1.2	Mahfoudhi *et al*., 2014
7	Luteolin	4.1	287.05479	1.4	Mahfoudhi *et al*., 2014
8	Quercetin	4.67	303.04968	1.5	Nawwar *et al*., 1984
9	Quercetin 3-sulfate	4.67	383.00659	1.6	Umbetova *et al*., 2005
10	Apigenin	7.28	271.05995	1.7	Ren *et al*., 2019
11	Chrysoeriol	9.42	301.0705	1.7	Xing *et al*., 2014
12	*N*-*trans*-Feruloyltyramine	5.12	314.13862	2.1	Orfali *et al*., 2009
13	Naringenin	1.53	273.07541	2.4	Karker *et al*., 2016

**Table 3:** List of the component’s metabolites of *T*. *nilotica* crude extract, which exhibit anti-cancerous activity generated from heatmap analysis. Retention time (RT), mass-to-charge ratio (m/z), and XlogP3 values are represented for each compound.

Compound name	RT	*m/z*	XlogP3	Reference
Citrate	0.47	193.03353	-1.7	Ren *et al*., 2017
Riboflavin	4.07	377.1456	-1.5	Mikkelsen *et al*., 2019
Rutin	4.89	611.16103	-1.3	Kim *et al*., 2005
Hallactone B	4.86	441.12158	-1.1	Russell *et al*., 1973
Dopamine	0.47	154.0863	-1	Lan *et al*., 2017
Orientin	5.06	449.10804	-0.2	Sharma *et al*., 2016
Apigenin-7-O-glucoside	5.53	433.11305	-0.1	Srivastava *et al*., 2007
Triptolide	6.12	361.16442	0.2	Wan *et al*., 2014
Biotin	4.83	245.09205	0.3	Tripodo *et al*., 2014
Epicatechin	3.86	291.08632	0.4	Abdulkhaleq *et al*., 2017
Betaine	0.37	118.08666	0.5	Sun *et al*., 2016
Gallic Acid	0.56	171.02821	0.7	PubChem
Isorhamnetin 3-glucoside	4.86	479.1186	0.7	Settu *et al*., 2017
Scutellarein-7-glucuronide	5.41	463.0871	0.8	Li NG *et al*., 2013
Quercetin 3,3-bissulfate	4.03	462.96351	1	Rauf *et al*., 2018; Hashemzaei *et al*., 2017
Caffeate	1.45	181.04928	1.2	PubChem
4-methylumbelliferone sulfate	5.11	257.01115	1.3	Yoshihara *et al*., 2005; Kudo *et al*., 2004
Luteolin	4.1	287.05479	1.4	Lin *et al*., 2008
6-Methoxyluteolin	4.37	317.06709	1.4	Chen *et al*., 2018
4-Hydroxybenzaldehyde	4.61	353.03269	1.4	Silke *et al*., 2016
Phenyl acetate	0.47	137.05977	1.5	PubChem
4-Coumarate	2.93	165.05449	1.5	Rosa *et al*., 2016
3-Hydroxy-4-methoxycinnamic acid	3.11	177.05445	1.5	Su *et al*., 2015
Quercetin	4.67	303.04968	1.5	Perez-Vizcaino *et al*., 2006
1-Acetoxyeugenol acetate	4.75	265.10684	1.5	Liew *et al*., 2017
4-Hydroxy-3-methoxycinnamaldehyde	5.27	179.06999	1.5	Thirunavukkarasu *et al*., 2018
4-hydroxy-3-methoxy-Cinnamic acid	7.13	209.08062	1.5	Ogiwara *et al*., 2002
Umbelliferone	3.79	163.03882	1.6	Mazimba, 2017
Hispidulin	7.49	301.07051	1.7	PubChem
4-Methylumbelliferone	6.77	177.05453	1.9	Lokeshwar *et al*., 2010
Fisetin	4.1	287.05479	2	Khan *et al*., 2008
Quinoline	4.82	130.06524	2	Jain *et al*., 2016
5,4_-Dihydroxy-7-methoxyflavone	9.27	285.0755	2.1	Li *et al*., 2017
Dihydroactinidiolide	8.03	181.12221	2.2	Das *et al*., 2018
Galangin	7.28	271.05995	2.3	Zhang *et al*., 2013
Naringenin	1.53	273.07541	2.4	Kanno *et al*., 2005
Tiliroside	6.64	595.14519	2.5	Tsimplouli *et al*., 2012
Carpacin	1.68	193.08569	2.7	Tseng *et al*., 2000
4-Isopropylbenzaldehyde	4.46	149.09605	2.7	Lee, 2005
Nevadensin	7.47	345.09686	2.9	Brahmachari *et al*., 2010
Isoliquiritigenin	5.08	257.08062	3.2	Bonesi *et al*., 2018
Desmethylxanthohumol	6.19	341.13812	4.7	PubChem
Stearidonic Acid	11.62	277.2162	5.2	Subedi *et al*., 2015
y-Linolenic acid	7.87	279.23169	5.9	Hrelia *et al*., 2010
Pygenic acid A b	14.59	473.36277	6.4	PubChem
Trans-Vaccenic acid	16.87	283.26309	6.5	Song *et al*., 2019
Stearic acid	0.23	285.27885	7.4	Habib *et al*., 1987
Lupenone	16.09	425.3778	9.6	Xu, 2018

### Solid phase fractionation of *T*. *nilotica* crude extract followed by MTT assay

The final objective of the study is to use a bio-guided fractionation approach to identify and obtain purified fractions that contain the bioactive compounds originally present in the complex crude *T*. *nilotica* extract. Solid phase extraction was performed to fractionate and isolate bioactive anti-cancer compounds in the *T*. *nilotica* crude extract. The crude extract was fractionated sequentially using different concentrations of methanol in water (10–100% v/v). Table 4 reports the cell viability (LD50; expressed as μg/mL) of *T*. *nilotica* methanol fractions, and the mass yield (%) of each fraction. The most bioactive fractions and the best yields were obtained with 10–70% methanol. The 30% and 70% (v/v) methanol fractions exhibited the highest anticancer activity against breast and lung cancer lines, respectively. The breast cancer cell line was more sensitive to the 30% methanol fraction (LD50 = 31 μg/mL) compared to the 70% methanol fraction (LD50 = 79 μg/mL). In contrast, colon cancer cells were more sensitive to the 70% methanol fraction (LD50 = 27 μg/mL) compared to the 30% methanol fraction (LD50 = 98 μg/mL).

**Table 4:** Cytotoxicity of the solid phase (C-18 column) fractionated aqueous extract of *T*. *nilotica* extract in methanol against breast (MDA-MB-2321) and colon (HCT-116) cancer cell lines. Yield of plant extracts and different fractions expressed as weight % of the original. Results are expressed as LD50 values (μg/mL). Values are means ± standard error of three experimental replicates.

Sample	% of methanol	Yields %	LD50 (μg/mL)		
			MDA-MB-231	HCT-116	
**Fr 1**	0	26.9	>100		>100
**Fr 2**	10	12.6	89 ± 0.008		>100
**Fr 3**	20	5.65	66 ±0.0035		97.7 ±0.004
**Fr 4** *	30	2.86	30.9 ±0.005		101 ±0.005
**Fr 5**	40	2.52	38.3 ±0.003		>100
**Fr 6**	50	1.67	85.2 ±0.012		>100
**Fr 7**	60	1.04	107 ±0.007		114.4 ±0.018
**Fr 8** *	70	0.618	78.8 ±0.002		27 ±0.04
**Fr 9**	80	0.147	>100		>100
**Fr 10**	90	0.0695	>100		>100
**Fr 11**	100	0.0445	>100		>100

* represent fractions with the highest inhibitory activity against cell lines.

**Fig 4:**
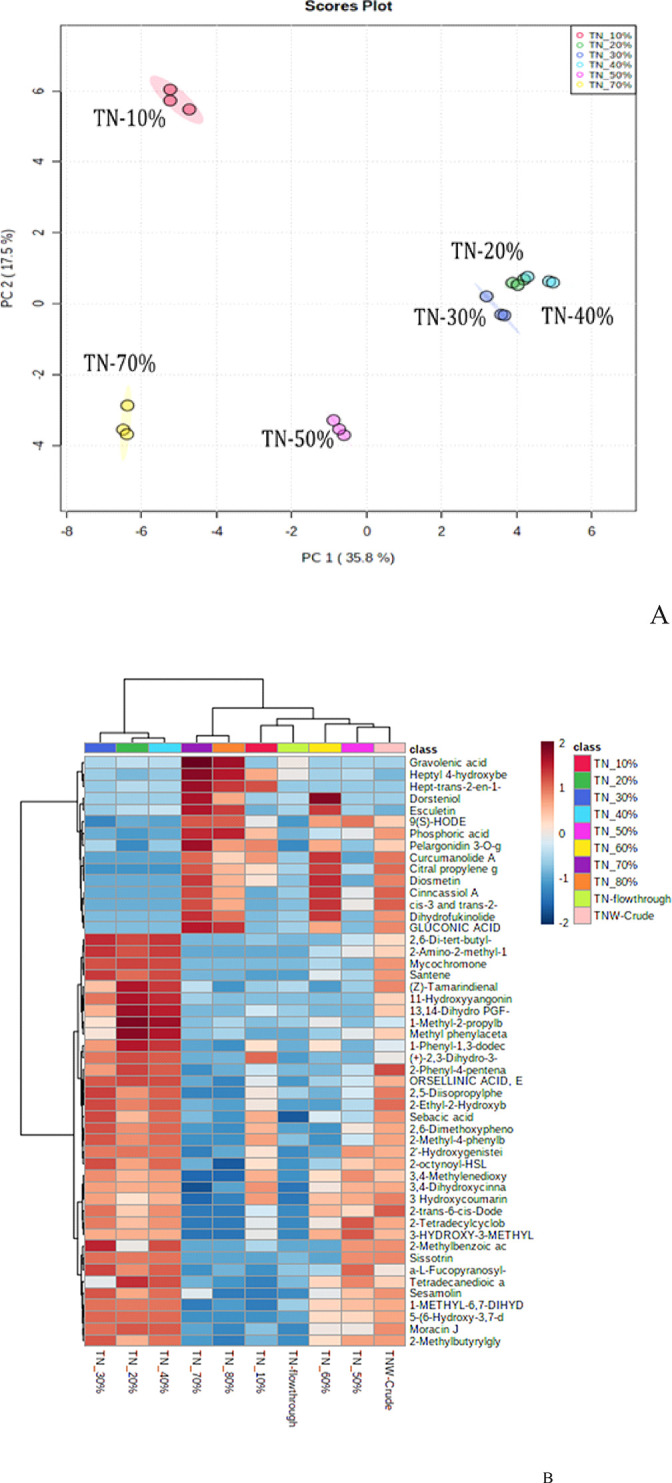
A) Graphical representation with 2-D scores of two principal components PC 1 and PC 2 comparing different methanol fractions of *Tamarix nilotica*. Each sample fraction has three experimental replicates. PCA generated after data log transformation and Pareto scaling. B) Heat map dendrogram plots of the metabolites analyzed by UPLCQ-TOF MS in crude extracts and different methanol fractions from *Tamarix nilotica*. Each sample has three experimental replicates. Heatmap plotted using Euclidean distance and clustering algorithm using ward.

Principle Component Analysis (PCA) was applied to investigate the differences between methanol fractions of *T*. *nilotica*, with a focus on the bioactive compounds in the 30% and 70% methanol fractions (Fig 4A). The PCA plot revealed that the fractions had distinct clusters based on their identity, with the 20%, 30%, and 40% methanol fractions in the same general area of distribution. However, the distribution of the 20% and 40% methanol fractions had some overlap with the 30% fraction, suggesting that the 30% fraction contains unique components that are not present in the other two fractions. This supports the bio-guided fractionation approach used in the study.

PCA plot showed the differences between methanol fractions of *T*. *nilotica*, with a particular focus on the bioactive compounds in the 30% and 70% methanol fractions. The PCA plot (Fig 4A) revealed that the 70% *T*. *nilotica* fraction had a distinct cluster separate from the other samples, suggesting that it has a significantly different chemical composition compared to the other *T*. *nilotica* fractions. The heatmap (Fig 4B) also shows substantial differences in chemical composition between the fractions. The study found that the 70% methanol fraction contains several compounds that have been associated with anticancer effects, including Estriol [48], Garbogiol [49], Hamamelitannin [50], and Gravolenic acid [51]. These compounds are believed to have contributed to the cytotoxicity observed in the 70% methanol fraction in the study. These results are consistent with findings reported by Mariane et. al. [52].

**Fig 5:**
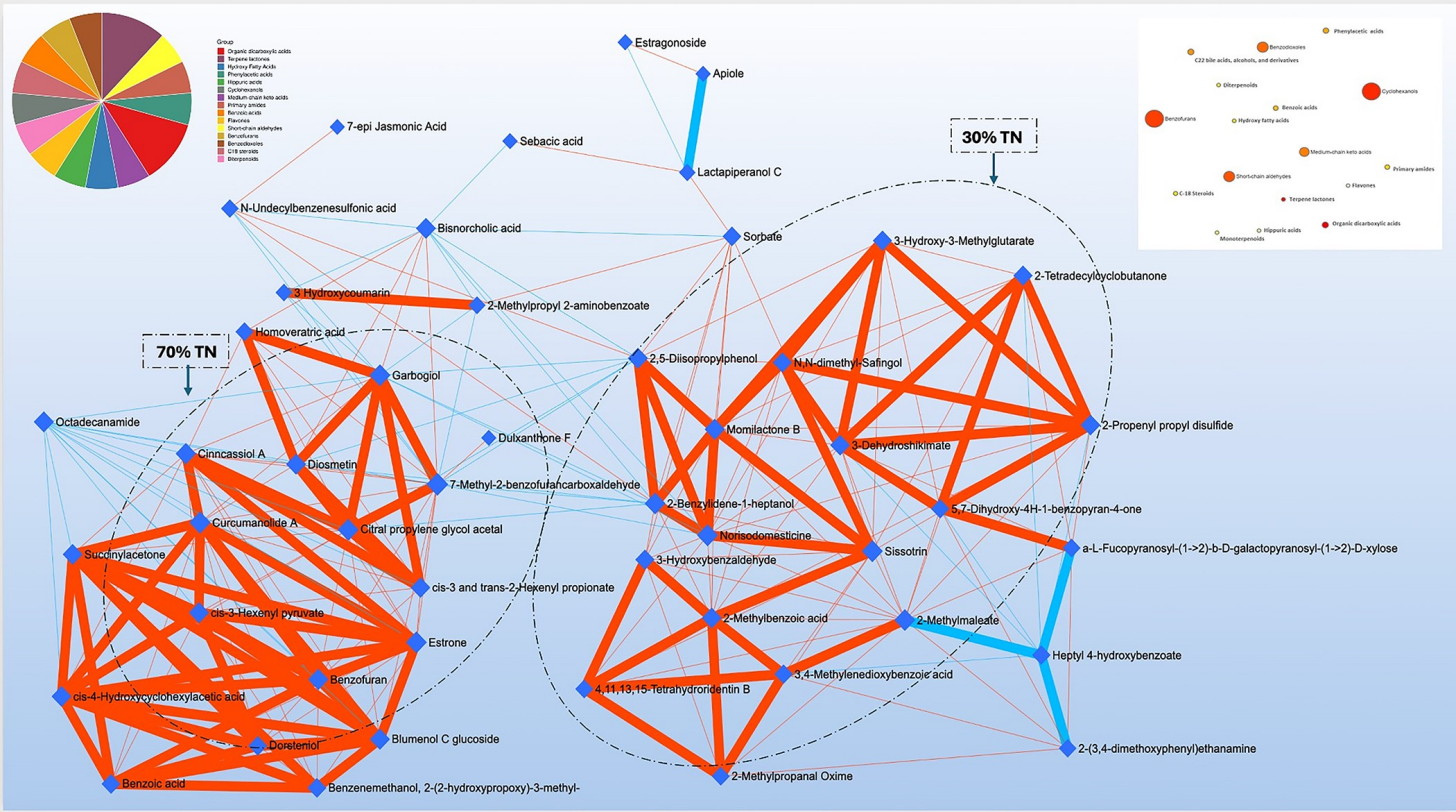
DSPC network for the top 50 annotated metabolic features in the 30%TN and 70%TN methanolic fractions. It represents prominent metabolic features in the 30% (right side) and 70% (left side) *T*. *nilotica* methanolic fractions with ellipse dashes. Nodes represent metabolites and edges depict the partial correlation coefficients or the associated p-values. The denser edges (width) red colour represents the strength of the associated metabolites with top correlations (partial correlation coefficient = 1 and p-value ≥ 0.01. The blue dense coloured edges are the metabolites strong evidence of negative relationship; partial correlation coefficient = -1. The light red and blue lines represent the low or zero degree of association or betweenness between compounds (See S4 Table). The two dashed ellipses highlight the top associated correlated compounds in each fraction.

To assess the biological relevance of the annotated metabolites to the anticancer effect, we applied the Debiased Sparse Partial Correlation (DSPC) network to reveal the relationship patterns among the most prominent metabolic features in the 30% and 70% *T*. *nilotica* methanolic fractions (Fig 5). The outcome can be visualized as weighted networks highlighting the most significant metabolic features based on VIP scores in the 30% (on the right side) and 70% (on the left side) methanolic fractions, using dashed ellipses for clarity. Within this network, nodes represent individual metabolites, and the connecting lines (edges) illustrate either the partial correlation coefficients or the corresponding p-values. The width and colour of the edges are used to convey information about the relationships between metabolites. Thicker and redder edges indicate strong positive correlations (where the partial correlation coefficient equals 1 and the p-value is greater than or equal to 0.01). Conversely, thicker, and bluer edges represent metabolites that have a strong negative relationship (with a partial correlation coefficient of -1). The lighter red and blue lines, on the other hand, represent metabolites that have low or no betweenness, suggesting limited influence or involvement with the compounds outside the dashed ellipses. For more detailed information, (See S4 Table).

The DSPC network revealed two main clusters of metabolites, with edges displaying a denser red colour (width) representing the strength of the associated metabolites with higher correlation. Many of the compounds in proximity shared metabolic pathway connections and chemical class similarities. Further examination was carried out to determine whether the strongest edges of the DSPC network were supported by biochemical evidence. Upon examining the left side of the network, it was observed that all the significant associated metabolites (p-value and correlation coefficient) were found in higher concentrations in the 70% TN fraction and included terpene lactones (curcumanose A; cinncassiol A), phenylacetic acids (homoveratric acid), cyclohexanols (cis-4-hydroxycyclohexylacetic acid), medium-chain ketone acids (succinylacetone) which were confirmed by the enrichment network pathway and pie-chart analysis.

The right side of the network showed that certain metabolites were present in high relative abundance in the 30% TN sample. These included benzoic acids, isoflavonoids, 3-hydroxybenzaldehyde, 3,4-methylenedioxybenzoic acid and 2-methylpropanol oxime. Similar compounds were found in Australian propolis, a by-product of honeybees, and have been shown to have anti-proliferative effects on certain cancer cells; MCF7 and the MDA-MB-231 [53]. These key metabolites were grouped into seven chemical categories, including 14 flavonoids, 7 triterpenes, and 6 acid derivatives such as quinic, coumaric, cinnamic, hydroxy-cinnamic, and hydroxy-benzoic. The cytotoxic effects of both the 70% TN and 30% TN fractions were likely due to a combination of various chemical compounds. These include phenylacetic acids, terpenes, benzofurans, isoflavonoids, and benzoic acids. For example, the synergistic effects of terpenes, phenylacetic acids, and benzoic acids have been shown to have antioxidant properties that can disrupt cell survival pathways, trigger apoptosis, and cause cytotoxic effects on colorectal cancer cells, which may support cancer treatment [54].

It has been observed that when studying metabolites, it’s not only important to look at individual compounds but also the combination of them [55,56]. Studies have shown that when metabolites are used together, they can have synergistic effects. This means that their combined effect is greater than the sum of their individual effects. For example, several studies have reported that using whole essential oils, which are a mixture of different volatile compounds, has been found to have greater activity against microorganisms such as L. *monocytogenes* and S. *enteritidis* than when using the major compounds alone [57]. Another study found that combining cinnamaldehyde with thymol or carvacrol had synergistic effects against *S*. *typhimirium*, with the results supporting two different hypotheses. One of the mechanisms proposed was that combining cinnamaldehyde with thymol or carvacrol could have altered the membrane permeability, allowing easy entry into the cell [58]. Another proposed that by binding with cell membrane proteins, this combination could have increased the number, size, or duration of the pores in the cell membrane. Essential oils constituents (i.e., phenolic component) are used as natural antimicrobial acting as membrane permeabilizers compounds reducing the impact of microbial activity in food products [59,60]. Therefore, it’s important to consider the potential interactions between metabolites when interpreting the results of a metabolomics study.

The DSPC method is a powerful tool in the field of metabolomics, it can be used to identify redundant features, unknown compounds, and discover the connectivity between metabolites in situations where pathway information is not readily available. Previous studies have also used this method to identify unknown compounds and discover the connectivity between metabolites using partial correlation networks [61–67]. Most high-scoring edges in partial correlation networks could be explained by pathway information and underlying biochemical knowledge, according to these studies. This method and tools can also help in identifying unknown features that are strongly linked to known metabolites. A high level of agreement between data-driven and knowledge-based biochemical networks is encouraging because it provides a foundation for identifying new connections between metabolites that may represent previously unknown metabolic regulatory interactions. This can help to provide a more complete picture of cellular metabolism.

## Conclusion

This study evaluated the potential use of two UAE halophytes (*T*. *nilotica* and *A*. *macrostachyum*) as a source of natural drugs for cancer treatment. The study revealed that; (1) the extraction method used to recover phytochemicals from plant biomass is essential, and MAE was found to be superior to conventional maceration extraction based on higher TPC, antioxidant capacity, and cytotoxicity effects of extracts. (2) Among the two halophyte species examined, only *T*. *nilotica* exhibited cytotoxicity towards breast, colon, and lung cancer cell lines. (3) Cancer cell lines exhibited various sensitivities to the *T*. *nilotica* extract, breast cancer being the most sensitive and lung cancer being the least sensitive to the cytotoxic effects of plant extract. (4) Annotated molecular features from the metabolomics profile of *T*. *nilotica* extract confirm the presence of several molecules reported by other studies to exhibit anticancer activity, such as terpenoids, flavonoids, and coumarins. (5) The successive fractionation of *T*. *nilotica* crude extract with increasing concentration of methanol in water revealed the 30% and 70% methanol fractions to be most bioactive (LD50 < 100 μg/mL).

This study explored *T*. *nilotica* halophyte as a promising natural source for discovering potentially novel chemo-preventive or chemotherapeutic drugs. Future work will focus on carrying out bio-guided fractionation steps of the 30% and 70% methanol fractions until a small number of molecules are detected. In addition, further analytical investigations should be carried out to identify and determine the structure of the non-annotated compounds along with biochemical experiments in vitro or in silico using molecular docking assays if a biological target is specified and its crystal structure is available.

## Materials and methods

### Plant collection and extraction

The study was conducted in accordance with relevant institutional, national, and international guidelines and legislation and none of the plants are at risk of extinction or on an endangered list. Fresh shoots of *Tamarix nilotica* and *Arthrocnemum macrostachyum* plants were collected from different zones of Abu Dhabi city. LF Yousef, a plant and soil scientist, identified the plants in consultation with botanists at the Environment Agency in Abu Dhabi. The identification process involved morphological characterization and consideration of the known geographic range of these plants in the UAE. Shoots of *Arthrocnemum macrostachyum* and *Tamarix Nilotica* were collected from a coastal desert location in Abu Dhabi, United Arab Emirates, in July 2016 (24°23’56"N 54°30’29"E) and voucher specimens were deposited in a herbarium hosted by the Environment Agency of Abu Dhabi. The herbarium is registered with Index Herbarium (http://sweetgum.nybg.org/science/ih/); herbarium code AED. Voucher specimen numbers for *A*. *macrostachyum* and *T*. *nilotica* are TERCO1670 and EAD3236, respectively. Plant shoots were washed with water and dried in an oven at 40°C for 48 hours. Dried plant biomass was then milled into a fine powder using a coffee grinder (50g/180W/MOULINEX-AR110O27) and stored at 22°C in an airtight plastic bag until used in metabolite extraction protocols.

Extraction of plant metabolites from plant biomass was carried out using two methods, Maceration and Microwave-Assisted Extraction (MAE). Powdered biomass from each plant (3g) was sterilized with milli-Q water (100 ml) in a glass bottle. For maceration extraction, a magnetic stir bar was placed in the glass bottle, and the sample was left to stir at room temperature for 24 hours. For MAE, a household microwave oven was used, and the sealed bottles were microwaved for three cycles at 30, 10, and 5 seconds. The temperature of the sample after each cycle did not exceed 60°C. Macerated and MAE extracts were filtered using Whatman GF/C paper, and the filtrates were concentrated in a rotary evaporator (IKA RV 10 digital V) at 40°C prior to lyophilization at -100°C and 0.250 Pa for 48 hours. Lyophilized extracts were stored at -80°C until further use, whereas extraction residues retained on the filter were dried in an oven to determine crude extract yields.

### Fractionation of crude plant extracts

Solid Phase Extraction (SPE) was performed to fractionate crude MAE extracts. Lyophilized crude extracts were dissolved in water and subjected to fractionation using C-18 SPE cartridges (WAT36905 – 6CC, 1g) that were first pre-conditioned with methanol. Crude extracts were loaded and sequentially eluted with an increased concentration of aqueous methanol solvent (10% to 100% methanol). Collected fractions were lyophilized at -100°C and 0.250 Pa for 48 hours, using pre-weighted collection tubes to determine fraction mass yields reported in Table 4 as Yield %.

### Total phenolic content (TPC)

According to Singleton and Rossi’s method, total phenolic content (TPC) was carried out using the Folin–Ciocalteu reagent with some modifications [68]. Gallic acid was used as a standard, and the standard curve (from 0.01 to 0.1 mg/ml diluted in methanol) was generated at 765 nm (Appendix B). Briefly, 0.5 ml of sample extract (from 1mg/ml stock solution) was added to 2.5 ml 10% Folin-Ciocalteu reagent and 2 ml of 7.5% Na_2_CO_3_ solutions vortexed and incubated in the dark for one hour at room temperature before absorbance measurement at 765 nm. Results were expressed as milligrams of Gallic acid equivalent (GAE) per g dry extract (mg GAE/g dry extract). The experiments were performed for *T*. *nilotica* and *A*. *macrostachyum* crude extracts in triplicate.

### Antioxidant capacity of extracts

1,1, Diphenyl-2-Picrylhydrazyl (DPPH) assay was performed to measure the antioxidant capacity of extracts. DPPH assay was carried out using a dilution series of plant extract (0.6–0.02 mg/ml). Briefly, 0.5 ml of each concentration was mixed with 0.3 ml of 0.5 mM DPPH and 3.0 ml of methanol, whereas the control consists of 0.3 ml of 0.5 mM DPPH and 3.5 ml of methanol only. The assay samples were kept in the dark at room temperature for 30 minutes before absorbance measurement at 517 nm. The absorbance data collected from these experiments were used to determine the IC50 value, which is the concentration of extract that scavenged 50% of the DPPH radical. The equation below represents the calculation of % DPPH free radical scavenging:

$$DPPH\;scavenging\;effects\;(\%)=\frac{Absorbance\;of\;control-Absorbance\;of\;sample}{Absorbance\;of\;control}\times 100$$

### Cell culture and cell cytotoxicity assay (MTT assay)

The cell lines used in this study were purchased directly from the American Tissue Culture Collection (ATCC). Three different carcinoma cell lines–MDA-MB231 (human breast cancer, ATCC#: HTB-26), HCT-116 (Human Colorectal Adenocarcinoma cancer, ATCC# CCL-247), and A549 (Human Lung adenocarcinoma cancer, ATCC# CCL-185) were used for *in vitro* cytotoxicity screening of plants extracts via MTT assays. The different cancer cell lines were plated in 96-well (cell-grade) plates at a density of 10–20 × 10^3^ cells/well and incubated at 37°C for 24 hours. Then, fractions of extract were applied at various concentrations (μg/mL) dissolved in Phosphate-buffered saline (PBS) for 72 hours, and each concentration was repeated in triplicate.

After 72 hours cells incubation, 150 μL of MTT stock solution (pre-prepared at a concentration of 5 mg/ml) was added to each well and left to incubate at 37°C for 2 hours until purple formazan crystal precipitation formed. Afterward, 100 μL of dimethyl sulfoxide (DMSO) was added to each well to dissolve the formazan crystals, and absorbance was measured at 595 nm using a microplate spectrophotometer (Infinite M200 PRO). Results were expressed in terms of cell viability (%), and LD50 values (μg/mL) were determined from the cell viability data. Experiments were repeated independently in triplicate. The equation below represents the calculation of % cell viable:

$$Cells\;viable\;(\%)=\frac{Absorbance\;of\;sample-Absorbance\;of\;blank}{\;Absorbance\;of\;control-Absorbance\;of\;blank}\times 100$$

### Untargeted metabolomic analysis of crude plant extracts

Metabolomics of crude extracts was analyzed using an Agilent 1290 Ultra-High-Performance Liquid Chromatography (UHPLC) system coupled to a Bruker Impact II quadrupole time-of-flight mass spectrometer (Q-ToF-MS, Bruker, Germany) [69]. Briefly, metabolites were separated using a reversed-phase Eclipse Plus C18 column (50 mm × 2.1 mm ID) (Agilent, US). Chromatographic separation consisted of MilliQ-H2O + 0.2% formic acid (buffer A), and acetonitrile + 0.2% formic acid (Buffer B) at a flow rate of 0.4 mL/min. The gradient started with 90% A and 5% B, increasing over 18 min to 100% B and holding at 100% B for 2 minutes. The column was then washed with isopropanol for 3 minutes and allowed to equilibrate for 4 minutes at the initial conditions.

Detection was carried out in the negative ionization modes with the following parameters: mass range = 50–1300 m/z measured at 6 Hz; ESI source parameters: dry gas temperature = 220°C, dry gas flow = 10.0 L/min, nebulizer pressure = 2.2 bar, capillary voltage = 3000 V, end plate offset = 500 V; MS-ToF tuning parameters: funnel 1 RF = 150 Vpp, funnel 2 RF = 200 Vpp, isCID Energy = 0 eV, Hexapole RF = 50 Vpp, ion energy = 4.0 eV, low mass = 90 m/z, collision energy = 7.0 eV, pre-pulse storage = 5 μs.

### Data processing and analysis

Data were processed using Metaboscape 3.0 (Bruker, Bremen, Germany). Processing was conducted with the T-Rex3D algorithm with an intensity threshold of 500 intensity counts and a minimum peak length of 10 spectra. Spectra were lock mass calibrated (622.0.28 m/z), and features were only created if detected in a minimum of 3 samples. Molecular feature identification was based on LC-MS accurate mass. MS^2^ fragmentation data was analysed using the large-scale metabolite library of standards (MSMLS, IROA, US), measured inhouse, and the Bruker Personal library with a match factor > 750 (max 1000) was used for annotation.

Following the data obtained from LC-MS, chemometrics and statistical analysis were carried out on the raw data (See S1 and S2 Tables) the MetaboAnalyst 5.0 platform (www.metaboanalyst.ca). The file input is a table with sample name, features name (*m/z*), and the peak abundance. The data integrity checks defaulted, no data filtering was conducted, and normalization was performed by log transformation and Pareto scaling data. Statistical analysis was performed using univariate (T-test), multivariate (principal components analysis–PCA), and unsupervised multivariate (heatmap cluster).

### Debiased sparse partial correlation (DSPC) network analysis

DSPC method was performed in conjunction with a correlation calculator “Metscape” to identify unknown compounds, undiscovered connectivity between metabolites and to remove redundant features (See S3 Table). In addition, by removing indirect connections, it can provide a more accurate understanding of the direct associations between metabolites following these steps: (i) Collect and pre-process the metabolomics data: Prepare the data for analysis by removing any outliers or missing values and normalizing the data. (ii) Use the correlation calculator to calculate the pairwise correlation coefficients between all the features in the data. (iii) Use the DSPC method to identify the strongest direct associations between features by removing indirect connections. (iv) Identify redundant features: Features that are highly correlated with one another can be considered redundant and removed from further analysis. (v) Identify unknown compounds: Features that are not strongly correlated with any other features can be considered unknown compounds and further investigated. (vi) Discover the connectivity between metabolites: Using the strongest direct associations identified by the DSPC method, a network of connections between metabolites can be constructed.

## Supporting information

S1 Table Excel sheet that contains tabs that contain raw data from the UHPLC-Q-ToF-MS analysis performed in this study. (XLSX)

S2 Table Raw data table containing the chemical features and abundance of each feature for the *Tamarix nilotica* and *Arthrocnemum macrostachyum* plant extracts used in the study. (CSV)

S3 Table Output table following applying the Debiased Sparse Partial Correlation (DSPC) method in conjunction with a correlation calculator on the extract UHPLC-Q-ToF raw data in order to identify unknown compounds, undiscovered connectivity between metabolites and to remove redundant features. (CSV)

S4 Table Output table following applying DSPC network to reveal the relationship patterns among the most prominent metabolic features in the 30% and 70% *T*. *nilotica* methanolic fractions. (CSV)
